# P-1587. Risk factors for necrotizing pneumonia in mechanically-ventilated patients with severe COVID-19

**DOI:** 10.1093/ofid/ofaf695.1766

**Published:** 2026-01-11

**Authors:** Alicia Hidron, Carlos Agudelo, Isabel Ramirez-Sanchez, Diana Moncada, Pablo Villa, Sara Penagos, Jose Albarracin, Gisela De la Rosa

**Affiliations:** Universidad Pontificia Bolivariana; SURA EPS, Medellin, Antioquia, Colombia; Universidad de Antioquia, Hospital Pablo Tobon Uribe, Medellin, Antioquia, Colombia; Hospital Pablo Tobon Uribe, Medellin, Antioquia, Colombia; Hospital Pablo Tobon Uribe, Medellin, Antioquia, Colombia; Hospital Pablo Tobon Uribe, Medellin, Antioquia, Colombia; Hospital Pablo Tobon Uribe, Medellin, Antioquia, Colombia; Hospital Pablo Tobon Uribe, Medellin, Antioquia, Colombia

## Abstract

**Background:**

During the COVID-19 pandemic we observed a high incidence of necrotizing pneumonias (NP) among mechanically ventilated patients (MVP) with severe COVID-19 (42 of 936, 4.5% patients). NP was associated to an elevated mortality (71% compared to 44% in COVID-19 MVP without NP). Pulmonary embolisms (PE) were documented in 31% of patients overall and in 50% patients who underwent CT angiography, suggesting that thrombosis and subsequent lung infarctions could be associated with this complication. The objective of this study was to understand the underlying risk factors for the development of NP among MVP with severe COVID-19.

**Table 1.**
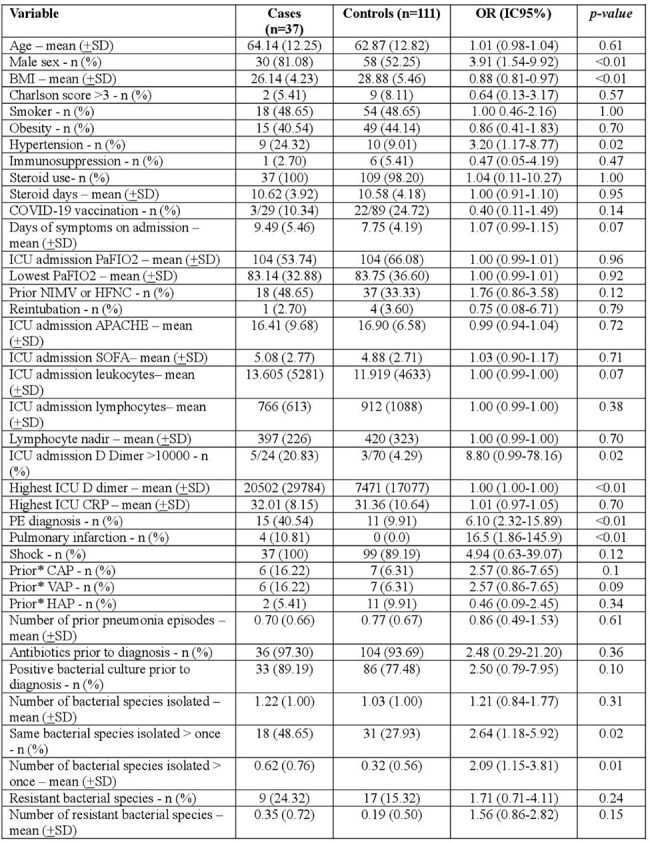
Risk factors for necrotizing pneumonia in mechanically-ventilated patients with severe COVID-19, univariate analysis ICU: intensive care unit, PaFIO2: ratio of arterial oxygen partial pressure (PaO2 in mmHg) to fractional inspired oxygen, CAP: community-acquired pneumonia, NIMV: non-invasive mechanical ventilation, HFNC: high-flow oxygen nasal cannula, PE: pulmonary embolism, CRP: C-reactive protein, VAP: ventilator-associated pneumonia, HAP: hospital-acquired pneumonia. *Prior (before diagnosis of necrotizing pneumonia for cases, during the period of observation for controls)

**Table 2.**
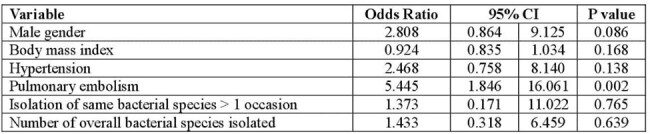
Risk factors for necrotizing pneumonia in mechanically-ventilated patients with severe COVID-19, multivariate analysis

**Methods:**

Case-control study including patients admitted to the intensive care unit (ICU) of a tertiary care hospital in Colombia for severe COVID-19, on mechanical ventilation (MV), between March 2020 and June 2021, matched by MV time +2 days. Demographic, clinical and microbiological data were collected. The univariate analysis was done with a conditional logistic regression including only the independent variable and the dependent variables, according to the matching group. Variables with a value of p< 0.05 were included in a conditional logistic regression model to identify factors associated with the development of NP. The study was approved by the ethics committee of hospital.

**Results:**

We included 37 cases and 111 controls. Male gender, a lower body mass index (BMI), hypertension (HTN), a higher D dimer on admission, and a confirmed diagnosis of PE were identified as risk factors for development of NP. Isolation of the same bacterial species from tracheal aspirates or BAL in more than one occasion, and the number of overall isolations prior to diagnosis of NP were also identified as risk factors. (Table 1) In the multivariate model, only a confirmed diagnosis of PE during the hospital stay, and isolation of the same bacterial species in more than one occasion prior to diagnosis, were found to be risk factors for the development of NP. (Table 2)

**Conclusion:**

Preceding PE and repeated isolation of the same bacterial species from tracheal aspirates or BAL in MVP patients with severe COVID-19 are associated to an increased risk of developing NP.

**Disclosures:**

All Authors: No reported disclosures

